# Nanoparticles Dual Targeting Both Myeloma Cells and Cancer-Associated Fibroblasts Simultaneously to Improve Multiple Myeloma Treatment

**DOI:** 10.3390/pharmaceutics13020274

**Published:** 2021-02-18

**Authors:** Honglan Wang, Huiwen Liu, Chunyan Sun, Chunying Liu, Ting Jiang, Yanxue Yin, Aoshuang Xu, Zhiqing Pang, Bo Zhang, Yu Hu

**Affiliations:** 1 Institute of Hematology, Union Hospital, Tongji Medical College, Huazhong University of Science & Technology, Wuhan 430022, China; d201881377@hust.edu.cn (H.W.); m201975631@hust.edu.cn (H.L.); 2006xh0838@hust.edu.cn (C.S.); 2018xh0213@hust.edu.cn (T.J.); 2018xh0215@hust.edu.cn (Y.Y.); xasjoanna@hust.edu.cn (A.X.); 2 Key Laboratory of Smart Drug Delivery, Ministry of Education, School of Pharmacy, Fudan University, 826 Zhangheng Road, Shanghai 201203, China; 19211030014@fudan.edu.cn

**Keywords:** multiple myeloma, CAFs, PDGFR-β, dual-targeting drug delivery, nanoparticles

## Abstract

Cancer-associated fibroblasts (CAFs) and myeloma cells could mutually drive myeloma progression, indicating that drug delivery to kill both CAFs and myeloma cells simultaneously could achieve better therapeutic benefits than to kill each cell type alone. Here, we designed a dual-targeting drug delivery system by conjugating paclitaxel (PTX)-loaded poly(ethylene glycol)-poly(lactic acid) nanoparticles (NPs) with a cyclic peptide (CNPs-PTX) with a special affinity with platelet-derived growth factor/platelet-derived growth factor receptor (PDGFR-β) overexpressed on both CAFs and myeloma cells. Cellular uptake experiments revealed that the cyclic peptide modification on CNPs could significantly enhance CNPs uptake by both CAFs and myeloma cells compared with unmodified NPs. Cytotoxicity tests showed that CNPs-PTX was more toxic to both CAFs and myeloma cells compared with its counterpart PTX-loaded conventional NPs (NPs-PTX). In vivo imaging and biodistribution experiments showed that CNPs could abundantly accumulate in tumors and were highly co-localized with CAFs and myeloma cells. The in vivo anti-tumor experiments confirmed that the anti-myeloma efficacy of CNPs-PTX was significantly stronger than that of NPs-PTX and free drugs. In summary, it is the first time that a dual-targeting strategy was utilized in the field of myeloma treatment through targeting both CAFs and myeloma cells simultaneously, which harbors a high potential of clinical translation for myeloma treatment.

## 1. Introduction

Multiple myeloma (MM), a malignancy of terminally differentiated plasma cells, is the second most common hematologic tumor. MM is still incurable despite exciting advances over the past few years by the application of different new strategies such as immunomodulatory drugs, proteasome inhibitors, and monoclonal antibodies, etc. [1,2]. Drug resistance and MM relapse represent a big challenge for myeloma treatment [3]. Accumulated evidence shows that the complex tumor microenvironment (TME), which serves as the soil for the myeloma cell surviving, is the main contributor to drug resistance, relapse, and progression of MM. TME and myeloma cells are always closely associated with each other and drive mutually to accelerate myeloma growth [4]. 

The TME of MM is mainly composed of extracellular matrix and many different types of stromal cells, including cancer-associated fibroblasts (CAFs), osteoclasts, and macrophages. As an important type of stromal cells in TME, CAFs have been reported to be closely related to tumor growth, metastasis, and invasion in different types of solid tumors. Some drugs targeting CAFs could modulate the TME and improve patient outcomes [5,6]. In MM patients, CAFs were closely associated with MM clinical stage and disease prognosis. CAFs could promote MM cell adhesion, proliferation and anti-apoptosis by secreting various cytokines and cell-to-cell interaction [7]. The interaction between CAFs and myeloma cells contributes to MM angiogenesis and progression [4], highly indicating that dual-targeting drug delivery to both CAFs (soil) and myeloma cells (seed) simultaneously could achieve a better therapeutic effect for MM treatment than targeting each cell type alone.

Nanotherapeutics could achieve a better therapeutic effect on tumors than free drugs based on the enhanced permeability and retention (EPR) effect of tumors [8,9]. However, the benefits of some anti-tumor nanotherapeutics, such as Doxil and Abraxane, two nanotherapeutics approved by the Food and Drug Administration (FDA) for use in solid tumors are still far from satisfactory [10,11]. As an extension, active targeting drug delivery systems, which are modified with targeting moieties holding special affinity to the corresponding antigens overexpressed in tumor tissues, have been explored to achieve additional anti-tumor efficacy compared with those based on the EPR effect alone [12,13]. Therefore, active drug delivery systems dual targeting both myeloma cells and CAFs simultaneously might achieve a strong therapeutic effect for MM treatment if suitable antigens overexpressed by both cells and corresponding targeting moieties can be selected.

Nowadays, it is widely accepted that the platelet-derived growth factor/platelet-derived growth factor receptors (PDGF/PDGFR) axis is involved in tumor angiogenesis, tumor growth, and metastasis [14]. PDGFR-β is low expressed in mesenchymal derived fibroblasts, vascular smooth muscle cells, monocytes, and other cell types under normal conditions. While in pathologic condition such as malignant tissue, PDGFR-β expression is strikingly increased in CAFs and tumor cells such as myeloma cells [6,15]. Moreover, the expression level of PDGFR-β is closely associated with MM angiogenesis and clinical stage [16], indicating that PDGFR-β could serve well as the therapeutic target for the dual-targeting strategy for myeloma treatment.

**Figure 1:**
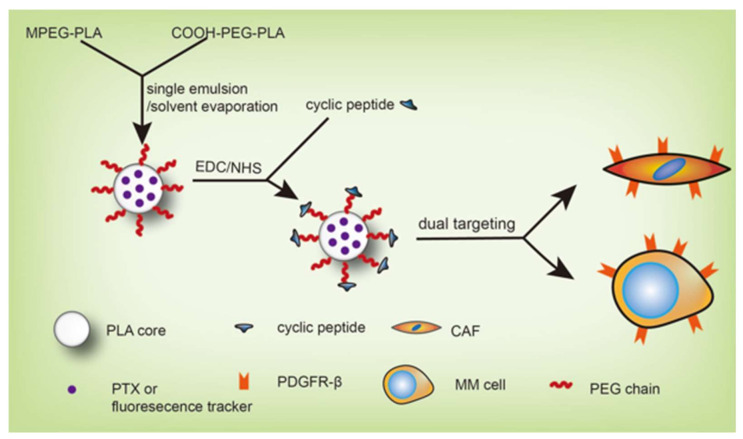
Schematic graph of dual-targeting drug delivery system design for myeloma treatment by targeting cancer-associated fibroblasts (CAFs) and myeloma cells simultaneously.

In the present study, a dual-targeting drug delivery system was designed by using a cyclic peptide with high affinity to PDGFR-β [5] as the targeting moiety, PDGFR-β overexpressed on both myeloma cells and CAFs as the therapeutic target, nanoparticles (NPs) based on FDA-approved biodegradable poly(ethylene glycol)-poly(lactic acid) as the drug carrier, and the classical chemotherapeutics paclitaxel (PTX) as the model drug (Figure 1). The cyclic peptide-modified NPs (CNPs) were synthesized and carefully characterized. The dual-targeting property of fluorescence-labeled CNPs was investigated by cellular uptake experiments, in vivo fluorescence imaging, and co-localization studies. The anti-myeloma effect of PTX-loaded CNPs (CNPs-PTX) was explored by cell apoptosis assay in vitro and an anti-myeloma test in vivo, and compared with those of Taxol and unmodified PTX-loaded NPs (NPs-PTX).

## 2. Materials and Methods

### 2.1. Materials

Cyclic peptide (Cys-Ser-Arg-Asn-Leu-lle-Asp-Cys (Cys&Cys bridge)) was synthesized by the Chinese Peptide Company (China). Methoxy-poly(ethylene glycol) (MPEG, MW 3000 Da) was ordered from NOF (Tokyo, Japan). R-carboxyl-poly(ethylene glycol) (COOH-PEG, Mw 3400 Da) was purchased from Laysan Bio (Arab, AL, USA). d,l-Lactide (purity: 99.5%) was from PURAC (Arkelsedijk, Holland). Methoxy-poly (ethylene glycol)-poly(lactic acid) (MPEG-PLA, Mw 33,000 Da) and R-carboxyl-poly(ethylene glycol)-poly(lactic acid) (COOH-PEG-PLA, Mw 33,400 Da) block copolymers were synthesized through ring-opening polymerization of lactide using MPEG and COOH-PEG as the initiator as previous reported [17]. Sodium cholate was purchased from Sigma (New York, NY, USA). Fluorescence tracker coumarin-6 was ordered from Sigma (New York, NY, USA). Hoechst 33342 was from Beyotime^®^ Biotechnology Co. Ltd. (Nantong, China). 1,1′-Dioctadecyl-3,3,3′,3′-tetramethylindo-tricarbocyanineiodide (DiR) was from Biotium (Invitrogen, Fremont, CA, USA). 1-(3-dimethylaminopropyl)-3-ethylcarbodiimide hydrochloride (EDC-HCl) and N-hydroxy-succinimide (NHS) were ordered from Sigma (New York, NY, USA). PTX was purchased from Xi’an San jiang Bio-Engineering Co. Ltd. (Xi’an, China). CD31 rabbit polyclonal primary antibody and CD138 monoclonal primary antibody were from Abcam (Cambridge, UK). α-Smooth muscle actin (α-SMA) rabbit monoclonal primary antibody was from Cell Signaling Technology (USA). Cy3-Affinipure goat anti-mouse IgG and Alexa fluor 647 Affinipure goat anti-rabbit IgG were obtained from Jackson (Philadelphia, PA, USA). The FITC (fluorescein isothiocyanate) Annexin V apoptosis detection kit I was purchased from BD Biosciences (San Jose, CA, USA). Fetal bovine serum (FBS), Newborn Calf Serum (NBCS), trypsin-EDTA (0.25%), RPMI 1640 medium, Modified Eagle’s Medium (MEM), and penicillin–streptomycin were from Gibco (Carlsbad, CA, USA). Plastic cell culture dishes were obtained from Corning Incorporation (Corning, NY, USA). Deionized water from the Millipore Simplicity System (Millipore, Bedford, MA, USA) was used throughout the entire study. All other reagents and chemicals were of analytical reagent grade and were purchased from Sinopharm Chemical Reagent (Shanghai, China).

RPMI 8226 cell lines were ordered from American Type Culture Collection. Mouse NIH-3T3 fibroblasts were ordered from the Chinese Academy of Sciences Cell Bank (Shanghai, China). They were cultured according to the recommended suggestions. Male Balb/c nude mice (six- to eight-week-old) were from the Shanghai Slac Lab Animal Ltd. (Shanghai, China). All experiments were carried out according to the regulations and standards of the ethics committee of Fudan University (approval code: 2017-03-YJ-PZQ-01,1 March 2017).

### 2.2. Synthesis and Characterization of CNPs

NPs were fabricated with a single emulsion/solvent evaporation method as previously described [8,18]. In a typical procedure, 1 mL of dichloromethane dissolving 24 mg of MPEG-PLA and 1mg COOH-PEG-PLA was added into 5 mL of 0.6% sodium cholate aqueous solution and then subjected to sonication (200 W, 5 s for 15 times) in an ice bath by a probe sonicator (Scientz Biotechnology Co. Ltd., Ningbo, China). After removing dichloromethane with a ZX-98 rotary evaporator (Shanghai Institute of Organic Chemistry, China), NPs was collected by centrifuge at 14,500 rpm for 45 min (Thermo Biofuge Stratos, Waltham, MA, USA). CNPs was prepared by reaction of carboxyl group on the surface of NPs with an amino group on cyclic peptide with EDC/NHS method as previously reported [18]. In our study, the molar ratio of the carboxyl group to amino group is 3:1, and the mass ratio of COOH-PEG-PLA to cyclic peptide is 1 mg: 20 µg.

Quantification of the cyclic peptide was performed by a high-performance liquid chromatography (HPLC) system (Agilent 1200 series; Santa Clara, CA, USA) equipped with an analytical column (150 mm × 4.6 mm; pore size 5 µm; ZORBAX 30 0SB-C18; Agilent). A mixture of solvent A (0.1% trifluoroacetic acid in water) and solvent B (80% acetonitrile solution containing 0.09% trifluoroacetic acid in water) in varying proportions was used as the mobile phase. Gradient program was increased linearly from 17% mobile phase B to 27% mobile phase B in 20 min at a flow rate of 1.0 mL/min. The sample injection volume was 10 µl, and the detector wavelength was 220 nm. The conjugation efficiency (CE) of cyclic peptide with NPs was calculated as follows:

(1) $$\mathrm{CE}=\frac{{\mathrm{cyclic}\;\mathrm{peptide}}_{\mathrm{total}}\;-{\;\mathrm{cyclic}\;\mathrm{peptide}}_{\mathrm{free}}}{{\;\mathrm{cyclic}\;\mathrm{peptide}}_{\mathrm{total}}}\times 100\%$$

The size distribution and zeta potential of NPs and CNPs were investigated by dynamic light scattering using a Malvern Nano ZS (Malvern Instruments, UK), and the morphology was observed using transmission emission microscopy (TEM) (H-600, Hitachi, Japan) after negative stained with 2% phosphotungstic acid. The stability of NPs and CNPs were investigated by monitoring different parameters including particle size, polydispersity index (PDI), and zeta potential during one-week storage in 5% FBS at 4 °C. 

Coumarin-6-, DiR-, and PTX-loaded NPs and CNPs were synthesized by the same method as blank NPs and CNPs except that 25 µg of coumarin-6, 200 µg of DiR, or 2 mg of PTX was added into the dissolved MPEG-PLA and COOH-PEG-PLA in 1 mL of dichloromethane in advance. 

### 2.3. Characterization of CNPs-PTX 

The drug-loading capacity (LC) and encapsulation efficiency (EE) and of NPs-PTX and CNPs-PTX were investigated as previously reported [18]. In brief, NPs-PTX or CNPs-PTX was dissolved in acetonitrile with the volume ratio of PTX-loaded NPs or CNPs to acetonitrile 1:9. After being vortexed for 1 min and centrifuged at 10,000 rpm for 10 min, the PTX concentration in the supernatant was measured by the HPLC system, as mentioned in the cyclic peptide concentration determination part. The mobile phase was a mixture of acetonitrile and water (CH3CN: H2O = 55:45, *v*/*v*) with a flow rate of 1.0 mL/min. The sample injection volume was 10 µl, and the detector wavelength was 227 nm. The LC and EE were calculated using Equations (2) and (3), respectively:

(2) $$\mathrm{LC}\;=\frac{\mathrm{Mp}}{\mathrm{Mn}}\times \;100\%\;$$

(3) $$\mathrm{EE}=\frac{\mathrm{Mp}}{\mathrm{Mpt}}\times 100\%$$

where Mp is the amount of PTX in the NPs, Mn is the amount of NPs, and Mpt is the total amount of PTX added to the formulation. 

The in vitro release behaviors of PTX from NPs-PTX and CNPs-PTX were investigated by a dialysis method [18]. Briefly, 1 mL of both PTX formulations with 60 µg of PTX was put into a dialysis bag (MWCO 8000 Da; Green Bird Inc, Shanghai, China) and incubated in 10 mL of release medium (0.0 1 M, pH = 7.4 PBS with 0.5% Tween-80) at 37 °C with the shaking speed of 120 rpm for 48 h. At each time point preset, 300 µl of aliquot was withdrawn, and fresh release medium with an equal volume was added immediately. The amount of PTX released at different time points was measured by the same method as mentioned above.

### 2.4. Cellular Uptake Experiment

RPMI 8226 cells were seeded into a laser confocal Petri dish at a density of 5 × 10^5^ cells/dish. After 24 h of culture, coumarin-6-labeled NPs and CNPs were added into the dish at a concentration of 20 ng/mL coumarin-6 per well and incubated at room temperature for 1 h. For the cyclic peptide inhibition experiment, excessive free cyclic peptide (10 µg/mL) was added 0.5 h before CNPs incubation. For the PDGFR inhibition experiment, PDGFR blocking agent suramin sodium (200 µmol/L) was added when CNPs incubation started [19]. Cells were subsequently washed three times with cold PBS (0.01 M, pH = 7.4), fixed with 4% paraformaldehyde, and then observed under fluorescence microscopy (Leica, Wetzlar, Germany). The semi-quantitative results were obtained by ImageJ using five randomly acquired images from different fields. NIH-3T3 cells were used to perform the same experiments as RPMI 8226 cells.

### 2.5. Apoptosis and CCK8 Assay

RPMI 8226 cells or NIH-3T3 cells were seeded into cell culture plates at a density of 5× 10^5^ cells/well. After 24 h of culture, different PTX formulations including Taxol, NPs-PTX, and CNPs-PTX were applied with 100 ng/mL PTX per well as the final concentration. Cells without any drug treatment were served as control group. After drug treatment for 24 h, cells from each group were collected and stained according to the Annexin V-FITC apoptosis detection kit and followed by quantitative analysis using flow cytometry (Beckman, Kraemer Boulevard Brea, CA 92821, USA). In addition, RPMI 8226 cells or NIH-3T3 cells were also seeded into 96-well cell culture plates at a density of 5 × 10^3^ cells/well; 24 h later, different PTX formulations including Taxol, NPs-PTX, and CNPs-PTX were applied with a series of PTX concentration from 1 to 200 ng/mL for 24 h culture. Then, the CCK8 reagent was added and incubated for 2 h. The absorbance at 420 nm of each well was measured by a microplate reader (Tecan Safire 2, männedorf, Switzerland) and IC50 were calculated by GraphPad Prism 7.0.

### 2.6. In Vivo Imaging

It has been previously reported that fibroblasts from MM patients could drive the proliferation of RPMI 8226 cells as compared with fibroblasts from healthy donors. RPMI 8226 cells could also promote the proliferation of fibroblasts. They found that MM cells and fibroblasts could interact with each other to drive the growth of myeloma [20]. To improve the success rate of deploying nude mice to develop MM mouse models and better simulate disease conditions, MM-bearing nude mice models were established by subcutaneous co-injection of 5 × 10^6^ RPMI 8226 cells and 2 × 10^6^ NIH-3T3 cells in 100 µL of PBS (0.01 M, pH = 7.4), which could consistently induce the formation of tumor xenograft within three weeks. As a comparison, nude mice that received the same amount of RPMI 8226 cells could not get an obvious xenograft until 4 weeks, and some even failed to induce the formation of xenograft (data not shown). When the diameter of the tumor reached 8 mm, the MM-bearing mice models received a tail vein injection of DiR-labeled NPs or CNPs with the dose of DiR 0.5 mg/kg. The mice models were imaged in vivo 24 h post DiR-labeled NPs administration by the In Vivo IVIS spectrum imaging system (PerkinElmer, USA). Then, 24 h post-drug administration, the mice were sacrificed and subjected to perfusion with 4% paraformaldehyde. Then, tumor tissues were collected, and the corresponding fluorescence signals ex vivo were also analyzed by the in vivo IVIS spectrum imaging system.

### 2.7. In Vivo Distribution 

MM-bearing nude mice models were injected with coumarin-6-labeled NPs and CNPs at the dose of coumarin-6 0.05 mg/kg. Then, 12 h later, the mice were sacrificed and perfused with 4% paraformaldehyde, and tumor tissues were prepared for frozen slices with a thickness of 20 µm. Immunofluorescence staining was performed as elsewhere described [9]. The vessels, MM tumor parenchymal cells, and CAFs were stained with CD31 rabbit polyclonal, CD138 rabbit monoclonal, and α-SMA rabbit monoclonal primary antibody (1:100) at 4 °C overnight respectively, and then further stained with Alexa fluor 647 Affinipure goat anti-rabbit IgG secondary antibody (1:100) for 1 h at room temperature. After the cell nucleus were stained by Hoechst 33342 (1 µg/mL) at room temperature for 10 min, the slices were mounted in Dako fluorescent mounting medium, captured under confocal microscopy (ZEISS, 710, LSM, wetzlar, Germany) and analyzed by ImageJ.

### 2.8. Anti-Myeloma Study In Vivo

MM-bearing nude mice models were established as described above. When the diameter of the tumor reached around 5 mm, the mice were randomly divided into four groups (*n* = 5), including the control group treated with saline, the Taxol group, the NPs-PTX group, and the CNPs-PTX group with the dose of PTX 5 mg/kg for each time. Treatment was continued five times every three days through tail vein administration. The tumor size and body weight were measured every three days during the experiment. The tumor volumes were calculated using the following formula:

(4) $$V=\frac{1}{2}\times a\times b^{2}$$

where a and b indicated the maximum and minimum diameters of the tumor, respectively. The tumor size and body weight were recorded for one more time after five cycles of treatments. When the whole experiment ended, tumors from all mice models were collected, and the weight of tumors was measured. Tumors were fixed with 4% paraformaldehyde, imbedded in paraffin, and sectioned at 5 µm for terminal dUTP-mediated nick-end-labeling (TUNEL) staining. The tumor slices of TUNEL staining were observed under the fluorescence microscope (Leica, Germany) at 200 × magnification, respectively. Tumor growth inhibition rates (TGIR) based on tumor size (TGIR_V_) and tumor weight (*TGIR_W_*) were calculated using the following formulas, respectively:

(5) $$TGIR_{V}=\frac{v_{c}-v_{t}}{v_{c}}$$

(6) $$TGIR_{V}=\frac{w_{c}-w_{t}}{w_{c}}.$$

In these formulas, *V_c_* and *V_t_* indicated the tumor volume in the control group and the treatment group, respectively; *W_c_* and *W_t_* indicated the tumor weight in the control group and the treatment group, respectively.

### 2.9. Statistical Analysis

All data were presented as mean ± SD (standard deviation). Statistical comparisons were performed with an unpaired Student’s t-test for two groups’ comparison. A probability (*p*) value < 0.05 was considered statistically significant.

## 3. Results and Discussions

### 3.1. Synthesis and Characterization of CNPs

**Figure 2:**
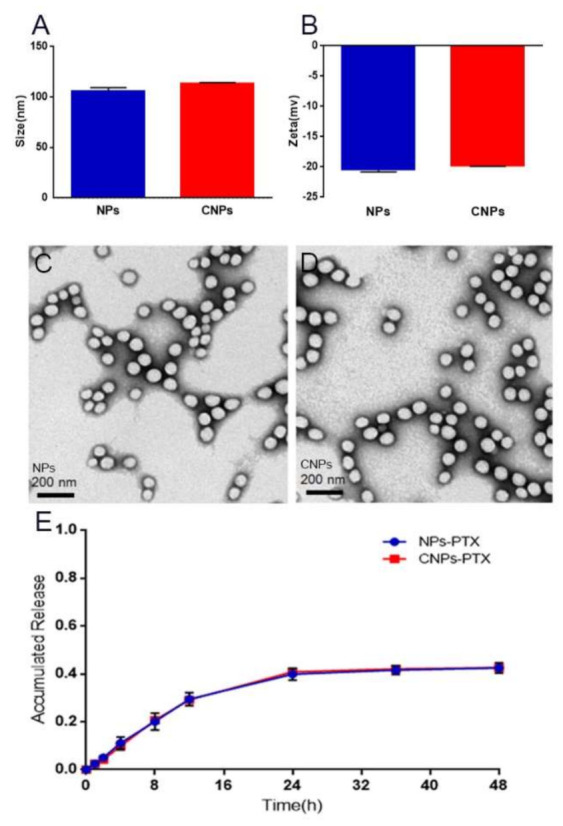
Characterization of cyclic peptide-modified nanoparticles (CNPs). Size distribution (**A**) and zeta potentials (**B**) of NPs and CNPs. Representative TEM image of NPs (**C**) and CNPs (**D**) (scale bar: 200 nm). Paclitaxel (PTX) release curve in vitro (**E**).

To develop a dual-targeting drug delivery system using PDGFR-β in myeloma tissues as the therapeutic target, a cycle peptide with a special affinity for PDGFR-β [19,21] was selected as the targeting moiety in our study, which is usually relatively more stable than other types of targeting moieties such as antibodies or proteins with much higher molecular weight [13]. As shown in Figure 2, the particle sizes of NPs and CNPs were about 100 nm. The size and zeta potential of the NPs increased slightly after the cyclic peptide was attached. This is consistent with some previous reports, which also showed that peptide modification did not significantly affect the size and zeta potential of conventional polymers NPs [22]. Both NPs and CNPs were negatively charged.The negative charge was mainly because of the negative charge of carboxyl end groups from COOH-PEG-PLA [23]. In addition, the isoelectric point of the cyclic peptide is 4.59 and negatively charged. The cyclic peptide modification did not significantly affect the zeta potential of NPs, the reason for which might be the number of peptide moiety on the surface of NPs being around 107, which might not be sufficient to affect the zeta potential of nanoparticles [18]. The TEM photos showed that both NPs and CNPs were of smooth surface and regular size, and they were slightly smaller than those measured with a dynamic light scattering method (Figure 2C,D). No significant particle size changes were observed within a week after both NPs and CNPs were dispersed in 5% FBS and stored at 4 °C (Figure S1). Under our experimental conditions (molar ratio of the carboxyl group to amino group = 3:1, reaction time = 4 h), the concentration of free cyclic peptide unconjugated to nanoparticles were below the detection baseline by HPLC, which indicated that almost all the cyclic peptides have been successfully conjugated to nanoparticles. The encapsulation of coumarin-6, DIR, or PTX into NPs exerts a slight effect on the particle size and the zeta potential of both NPs and CNPs (Figure S2). 

The LC values of NPs-PTX and CNPs-PTX were 5.58 ± 0.01% (*n* = 3) and 4.05% ± 0.12 (*n* = 3), respectively. The EE values of PTX in NPs-PTX and CNPs-PTX were 37.62% ± 1.22 (*n* = 3) and 32.55% ± 0.92 (*n* = 3), respectively. The in vitro release profiles of PTX from both nanotherapeutics showed a similar biphasic pattern (Figure 2E), indicating that cyclic peptide modification did not significantly affect the in vitro release behavior of PTX from NPs. The sizes of these fluorescence tracker-labeled or drug-loaded NPs and CNPs are around 100 nm and fully meet the requirements of the drug delivery system for in vivo research as previously reported [18], which will be further used in subsequent experiments to assess its myeloma dual-targeting ability and anti-myeloma effect. 

### 3.2. Cellular Uptake Experiment 

**Figure 3:**
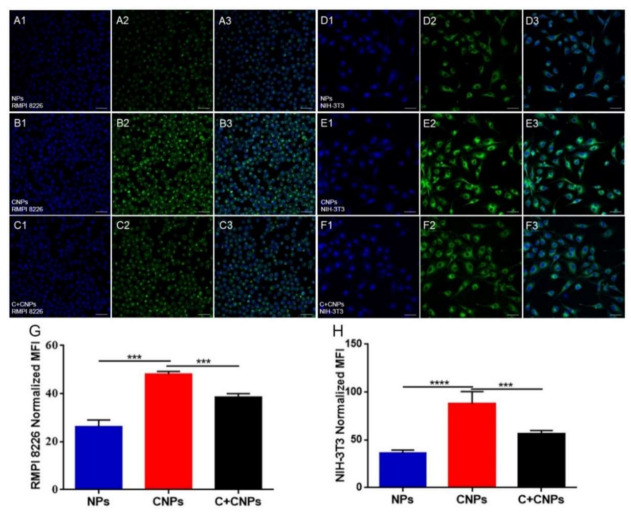
Cellular uptake experiments for RPMI 8226 cells (**A**–**C**,**G**) and NIH-3T3 cells (**D**–**F**,**H**). Fluorescence images for in vitro uptake of coumarin-6-labeled NPs (**A**,**D**), CNPs (**B**,**E**), and CNPs with free cyclic peptide preincubation (**C**,**F**), respectively. Image 3 was obtained by merging images 1 and 2. The corresponding semi-quantitative results (**G**,**H**) were obtained using Image J software. Green indicated coumarin-6 labeled NPs or CNPs. Blue indicated cell nuclei. Original magnification: 200×. *** *p* < 0.001, **** *p* < 0.0001.

To investigate the dual-targeting ability of CNPs, NIH-3T3 fibroblasts and RPMI 8226 myeloma cells, which have a high expression of PDGFR-β molecule as reported [16], were selected as in vitro cell models for cellular uptake experiments in the present study. As shown in Figure 3, the CNPs group showed a much stronger signal in both RPMI 8226 cells and NIH-3T3 cells than the NPs group. The cellular uptake of CNPs by both types of cells could be inhibited to a certain degree by incubation with free cyclic peptide in advance. In addition, the uptake of CNPs could also be inhibited by coculture with PDGFR blocking agent suramin sodium (Figure S3). The semi-quantitative result obtained by ImageJ showed a similar result. These results demonstrated that the cyclic peptide could improve the cellular uptake of NPs by both NIH-3T3 cells and RPMI 8226 cells, which is probably through the interaction between the cyclic peptide modified on the surface of NPs and PDGFR-β overexpressed by these two types of cells. The cellular uptake outcomes also indicated that drug-loaded CNPs might have higher cytotoxicity than the corresponding NP encapsulated with chemotherapeutic agents.

### 3.3. Apoptosis and CCK8 Assay 

**Figure 4:**
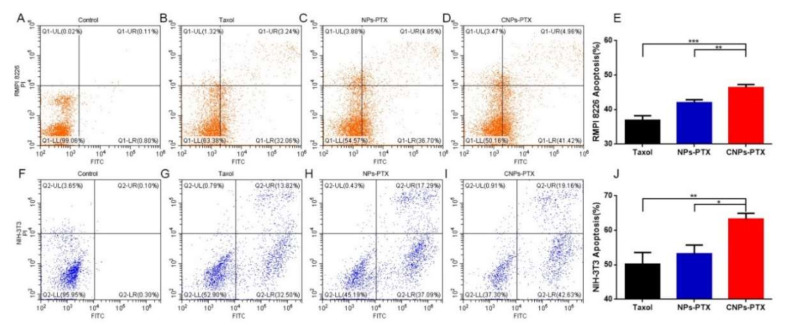
Cytotoxic assay. Representative flow cytometer images of RPMI 8226 cells and NIH-3T3 cells 24 h after different drug treatment Taxol (**B**,**G**), NPs-PTX (**C**,**H**), CNPs-PTX (**D**,**I**) at the dose of PTX 100 ng/mL. Samples without any drug treatment served as control (**A**,**F**). The flow cytometry quantitative results for RPMI 8226 cells (**E**) and NIH-3T3 cells (**J**). * *p* < 0.05, ** *p* < 0.01, *** *p* < 0.001.

To test our hypothesis mentioned above, classical chemotherapeutics PTX with favorable lipophilicity was used as the model drug, while the cytotoxic effect of different PTX formulations to RPMI 8226 cells and NIH-3T3 cells in vitro was determined by apoptosis and CCK8 assay. Different PTX formulations, including Taxol, NPs-PTX, and CNPs-PTX were added to the cells, incubated for 24 h, and followed by flow cytometry detection of cell apoptosis. The results showed that CNPs-PTX could induce more cell apoptosis than NPs-PTX and Taxol for both cell types (Figure 4). The total apoptosis rate of RPMI 8226 cells in the CNPs-PTX group is 46.36 ± 0.75%, which is much higher than 42.03 ± 0.70% and 36.83 ± 1.12% in NPs-PTX group and Taxol group, respectively. For NIH-3T3 cells, the apoptosis rate is 63.31 ± 1.36% in the CNPs-PTX group, which is also much higher than 53.23 ± 2.04% and 50.17 ± 2.19% in NPs-PTX group and Taxol group, respectively. The CCK8 assay showed that all PTX formulations exhibited inhibitory effects to the proliferation of RPMI 8226 cells and NIH-3T3 cells. At various concentration points, CNP-PTX exhibited the strongest inhibitory effect on both cells (Figure S4 and Table S1). The results of cytotoxicity assay were consistent to that of the apoptosis assay. All these results were consistent with the results of cellular uptake, indicating that cyclic peptide modification can enhance cellular uptake and therefore cytotoxicity, further confirming the dual-targeting ability of CNPs-PTX.

### 3.4. In Vivo Imaging 

**Figure 5:**
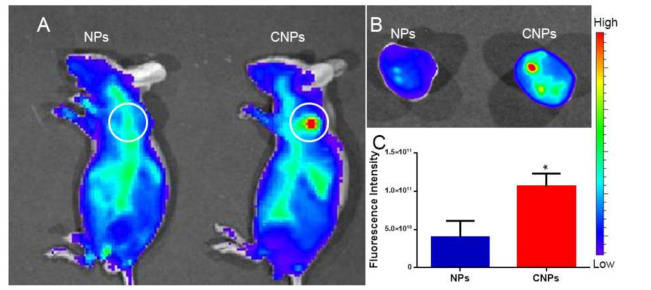
In vivo imaging. In vivo imaging of tumor-bearing mice 24 h after 1,1′-dioctadecyl-3,3,3′,3′-tetramethylindo-tricarbocyanineiodide (DiR)-loaded NPs and CNPs treatment (**A**). Ex vivo imaging of tumors (**B**) and the corresponding semi-quantitative result (**C**). White circle: tumor site. * *p* < 0.05.

In vivo imaging was performed to understand the targeting ability of CNPs to myeloma in vivo. The whole-body image acquired 24 h post-injection showed that the fluorescence signal intensity in the tumor site of the CNPs group was much higher than that of the NPs group (Figure 5A). Similarly, the fluorescence intensity of the tumor in the CNPs group ex vivo was also higher than that in the NPs group (Figure 5B), and the ex vivo semi-quantitative results showed that the fluorescence signal intensity of the tumor in the CNPs group was 2.3 times that of the NPs group (Figure 5C). 

### 3.5. In Vivo Distribution

**Figure 6:**
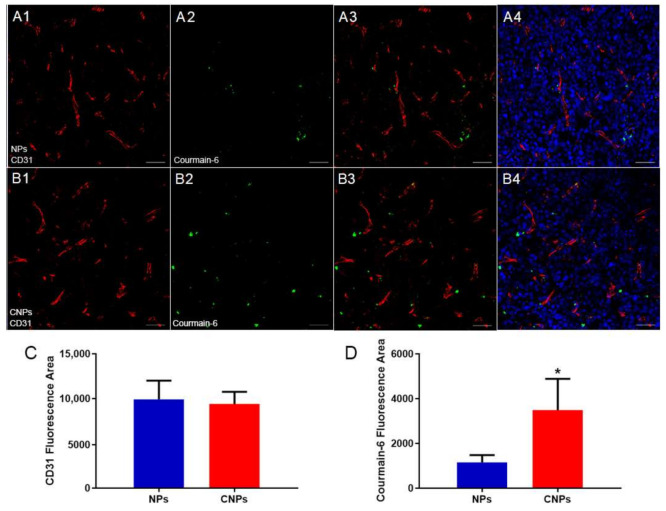
In vivo distribution experiment. The images of distribution of NPs (**A1**–**A4**) and CNPs (**B1**–**B4**) in tumor tissues 24 h post-injection of courmain-6 labeled NPs or CNPs. Image 3 was merged by images 1 and 2. Image 4 was merged by nucleus and image 3. Semiquantitative results of signal of vessels indicated by CD31 fluorescence area (**C**) and signal of NPs and CNPs indicated by coumarin-6 fluorescence area (**D**) analyzed by ImageJ (*n* = 3). * *p* < 0.05. Green indicated courmain-6-labeled NPs or CNPs. Red indicated CD31-labeled tumor vessels. Blue indicated cell nucleus. Original magnification: 200×. Scale bars, 50 µm.

**Figure 7:**
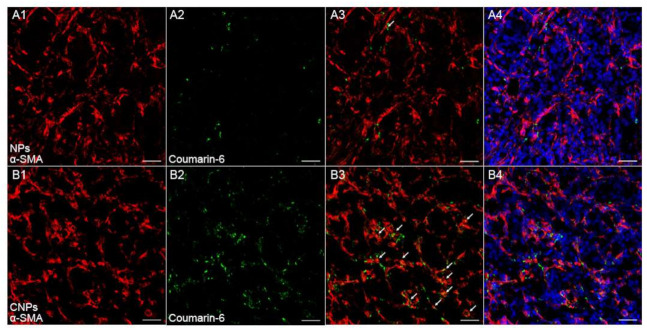
In vivo co-localization of NPs (**A**) or CNPs (**B**) with CAFs. Image 3 was the merged by images 1 and 2. Image 4 was merged by nucleus and image 3. Red indicated CAFs labeled by α-smooth muscle actin (α-SMA). Green indicated coumarin-6 labeled NPs or CNPs. Blue indicated cell nuclei. The arrow indicated the co-localization of coumarin-6 labeled NPs or CNPs and CAFs. Original magnification: 200×. Scale bars, 50 µm.

**Figure 8:**
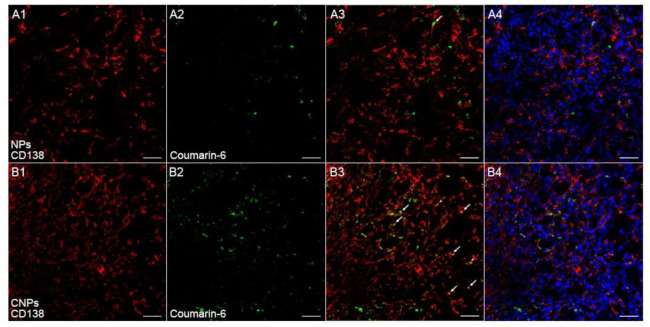
In vivo co-localization of NPs (**A**) or CNPs (**B**) with myeloma cells. Image 3 was created by merging images 1 and 2. Image 4 was created by merging the nucleus and image 3. Red indicated myeloma cells labeled by CD138. Green indicated coumarin-6-labeled NPs or CNPs. Blue indicated cell nuclei. The arrow indicated the co-localization of coumarin-6 labeled NPs or CNPs and myeloma cells. Original magnification: 200×. Scale bars, 50 µm.

Since the overall accumulation of nanotherapeutics in tumor tissues alone did not ensure optimal therapeutic efficacy, a homogeneous distribution pattern of nanotherapeutics within tumor tissues might be more critical for the final performance of nanotherapeutics [24,25]. Nanotherapeutics distribution within myeloma was detected by immunofluorescence staining followed by confocal microscopy analysis. To track NPs in vivo, green fluorescence tracker coumatin-6 was used to label NPs. Results of immunofluorescence staining showed only a few NPs localized in the vicinity of tumor vessels. In comparison, CNPs are not only abundantly deposited nearby tumor vessels but also penetrate tumor tissues to reach those regions far away from tumor vessels (Figure 6A,B); the quantitative evaluation was also performed to show that the signal from CNPs was much higher than that from conventional NPs (Figure 6D), while there was no significant difference between the NPs and CNPs group in regard to the signal from tumor vessels (Figure 6C), which suggested that CNPs could distribute more widely in tumor tissues than NPs. Furthermore, more CNPs were found to be colocalized with myeloma cells or CAFs than NPs (Figure 7, Figure 8 and Figure S5), which suggested that more CNPs could be uptaken by both CAFs and myeloma cells as compared with NPs. These results compared well to the results of in vitro uptake (Figure 3) and in vivo fluorescence imaging experiments (Figure 5), which altogether validated the dual-targeting ability of CNPs in vitro and in vivo. Therefore, more drugs can be delivered by CNPs to PDGFR-β overexpressed myeloma cells and CAFs, and better therapeutics benefit can be predicted when PTX was loaded.

### 3.6. Anti-Myeloma Study In Vivo

**Figure 9:**
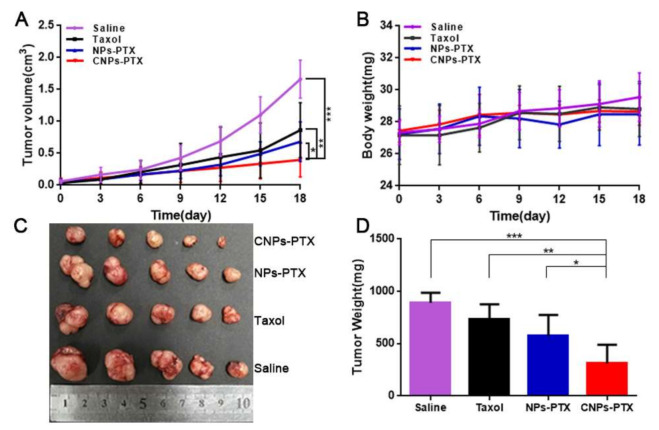
In vivo anti-myeloma efficacy of paclitaxel (PTX)-loaded poly(ethylene glycol)-poly(lactic acid) nanoparticles (NPs) with a cyclic peptide (CNPs-PTX). Tumor growth curve (**A**) and weight change curve of mice models during the experiment (**B**). Gross appearance of tumors (**C**) and tumor weight (**D**) at the study end point. * *p* < 0.05, ** *p* < 0.01, *** *p* < 0.001.

To understand the therapeutic efficacy of CNPs-PTX for myeloma treatment, a pharmacodynamics experiment was carried out. As shown in Figure 9A, the tumor growth was significantly inhibited in the CNPs-PTX group compared with the other groups. Taxol and NPs-PTX had only mild therapeutic effects compared with the control group. The *TGIRv* and *TGIRw* for the CNPs-PTX group were 64.9% and 76.5%, which was much higher than 35.5% and 41.1% in the NPs-PTX group and 17.8% and 29.0% in the Taxol group (Figure 9C,D). After five cycles of treatment, the tumors were removed for TUNEL staining. The green fluorescence was much more in the CNPs-PTX group than that of the other three groups (Figure S6), indicating that the most severe tumor cell apoptosis occurred in this group, which accorded well to the tumor inhibitive curve. There were no obvious changes in the body weight of mice models during the experiment (Figure 9B), indicating that the PTX dose in our study is safe for mice. Overall, the best anti-myeloma effect was found in the CNPs-PTX group, which is probably due to its dual-targeting ability to both CAFs and myeloma cells.

Varying degrees of success have been achieved for tumor drug delivery based on a dual-targeting strategy as previously reported. In one study, fusion protein EGFP-EGF1 with high affinity for tissue factor (TF) has been selected as the targeting moiety, and TF overexpressed by different cell types, including tumor parenchyma cells, TAFs, and tumor-associated endothelial in glioma and lung cancer, was utilized as the therapeutic target [13,26]. Some other studies also utilized two different targeting moieties to conquer different obstacles hindering tumor drug delivery [27,28]. These dual-targeting nanotherapeutics are less ideal because of the relative instability of high molecular protein and the synthetic complex of conjugating two different targeting moieties. As a comparison, our single cyclic peptide modified dual-targeting CNPs have the advantages of more stability of targeting moiety and synthetic simplicity of conjugating only a single targeting moiety.

This strategy opened a new avenue for myeloma treatment by dual targeting both CAFs (the soil) and myeloma cells (the seed) simultaneously. Since CAFs produce a lot of cytokines to drive myeloma progression and are closely associated with a tumor immune inhibitive state [20,29], the changes of immune state within a myeloma site deserves further study to understand whether the therapeutic effect was immune-related or not, which requires a mouse-derived MM cell line and immune competent recipient mice to develop a suitable mouse model. In addition, PDGFR-β was also overexpressed in various other types of tumor parenchymal cells such as breast cancer cells and colon carcinoma cells [6,15,30]; the dual-targeting nanotherapeutics modified with the cyclic peptide might provide a new avenue for the treatment of other solid tumors as well.

## 4. Conclusions

In this study, we exploited a cyclic peptide with high affinity with PDGFR-β as a targeting moiety to establish a dual-targeting drug delivery system that could not only improve the cellular uptake of CNPs by myeloma cells and CAFs but also induce more cell apoptosis of both types of cells when PTX was loaded. In vivo tests showed dual-targeting CNPs exhibited significantly stronger myeloma accumulation, myeloma targeting, and enhanced chemotherapeutic effect of PTX as compared with those of other groups in myeloma-bearing mouse models. Altogether, it is the first time that a dual-targeting strategy was applied for myeloma treatment, and these promising outcomes suggested that the dual-targeting drug delivery system might exhibit great potential for myeloma treatment in clinical applications.

## Data Availability

The data presented in this study are available in Supplementary Materials: https://www.mdpi.com/1999-4923/13/2/274/s1.

## Supplementary Materials

The following are available online at https://www.mdpi.com/1999-4923/13/2/274/s1, Figure S1: Size (A) and PDI (B) of NPs and CNPs in 10% FBS during one-week storage at 4 °C. (*n* = 3), Figure S2: Characterization of different types of NPs and CNPs. A. Size of Coumarin-6-, DiR- and PTX-loaded NPs and CNPs. B. Zeta potential of Coumarin-6-, DiR- and PTX-loaded NPs and CNPs. (*n* = 3), Figure S3: CNPs uptake by RPMI 8226 cells (A,B,C) and NIH-3T3 cells (D,E,F) treated with (B&E) or without (A&D) PDGFR-β blocking agent suramin sodium analyzed by microscopy (A,B,D,E) and ImageJ (C&F). Green indicated coumarin-6 labeled CNPs. Blue indicated cell nuclei. Original magnification: 200×, Figure S4: In vitro cytotoxicy of Taxol, NP-PTX and CNP-PTX against RPMI 8226 cells (A) and NIH-3T3 cells (B) by CCK8 assay, Figure S5: In vivo co-localized study of coumarin-6 labeled NPs (A,E,C,G) or CNPs (B,F,D,H) with fibroblasts (C,G,D,H) or myeloma cells (A,E,B,F) observed by confocal (A,B,C,D) and analyzed by ImageJ and origin (E,F,G,H). The signal intensity in the histogram is obtained from the line randomly put in the corresponding figure, Figure S6. TUNEL staining of tumor tissue sections from MM bearing mice model of in vivo anti-myeloma study (left to right: Saline, Taxol, NPs-PTX, and CNPs-PTX). Table S1: IC50 of RPMI8226 and NIH-3T3 by Taxol, NP-PTX and CNP-PTX calculated by GraphPad Prism 7.0 based on CCK8 assay.
