# Supplementary material for: Nanoparticles Dual Targeting Both Myeloma Cells and Cancer-Associated Fibroblasts Simultaneously to Improve Multiple Myeloma Treatment
Source: Pharmaceutics. 2021 Feb 18;13(2):274. doi: 10.3390/pharmaceutics13020274 (PMC7922689; doi:10.3390/pharmaceutics13020274)
Supplement: Supplementary file 1 [file pharmaceutics-13-00274-s001.pdf]

pharmaceutics MDPI

Supplementary Materials: Nanoparticles Dual Targeting Both Myeloma Cells and Cancer-Associated Fibroblasts Simultaneously to Improve Multiple Myeloma Treatment

Honglan Wang [1], Huiwen Liu [1], Chunyan Sun [1], Chunying Liu [2], Ting Jiang [1], Yanxue Yin [1], Aoshuang Xu [1], Zhiqing Pang [2,*], Bo Zhang [1,*] and Yu Hu [1,*]

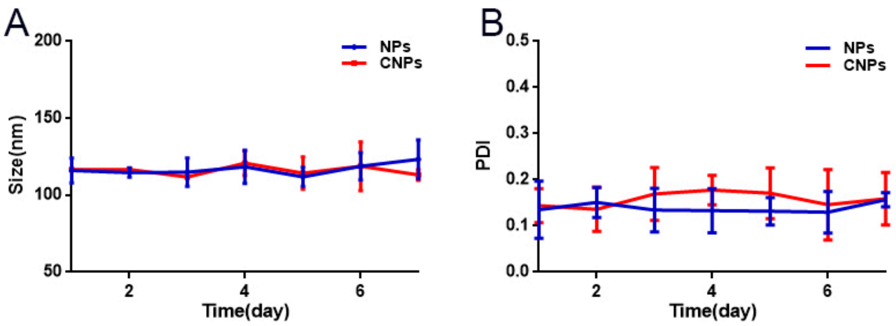

Figure S1. Size (A) and PDI (B) of NPs and CNPs in 10% FBS during one-week storage at 4 °C. (n = 3).

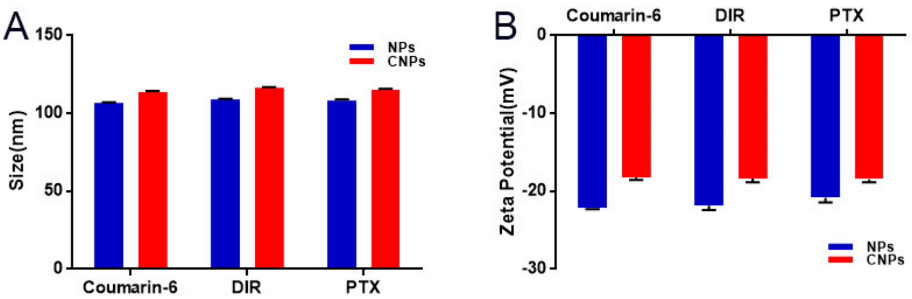

Figure S2. Characterization of different types of NPs and CNPs. A. Size of Coumarin-6-, DiR- and PTX-loaded NPs and CNPs. B. Zeta potential of Coumarin-6-, DiR- and PTX-loaded NPs and CNPs. (n=3).

Publisher's Note: MDPI stays neutral with regard to jurisdictional claims in published maps and institutional affiliations.

Copyright: © 2020 by the authors. Submitted for possible open access publication under the terms and conditions of the Creative Commons Attribution (CC BY) license (http://creativecommons.org/licenses/by/4.0/).

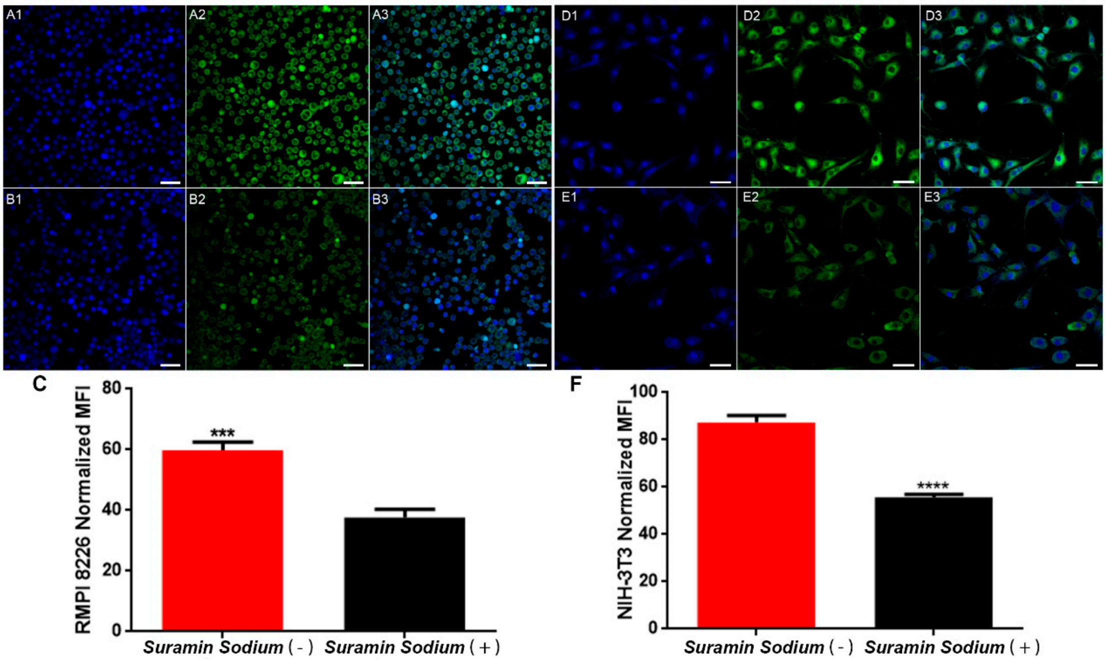

Figure S3. CNPs uptake by RPMI 8226 cells (A, B, C) and NIH-3T3 cells (D, E, F) treated with (B & E) or without (A & D) PDGFR-β blocking agent suramin sodium analyzed by microscopy (A, B, D, E) and ImageJ (C & F). Green indicated coumarin-6 labeled CNPs. Blue indicated cell nuclei. Original magnification: 200×.

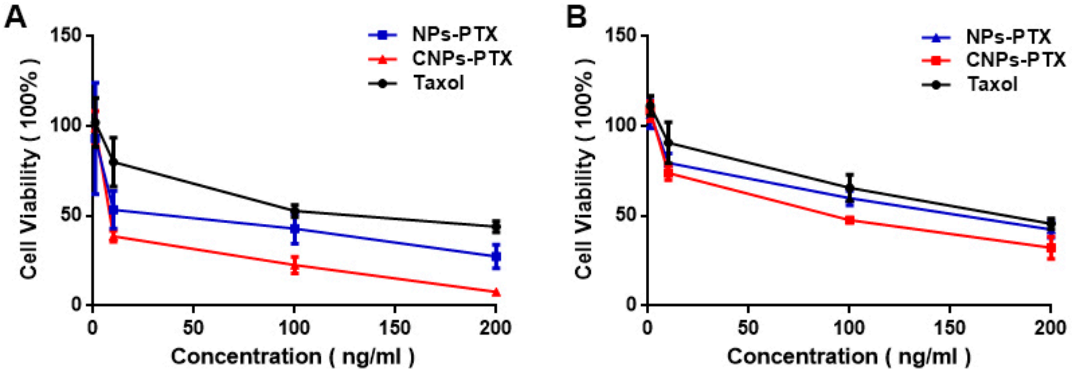

Figure S4. *In vitro* cytotoxicity of Taxol, NP-PTX and CNP-PTX against RPMI 8226 cells (A) and NIH-3T3 cells (B) by CCK8 assay.

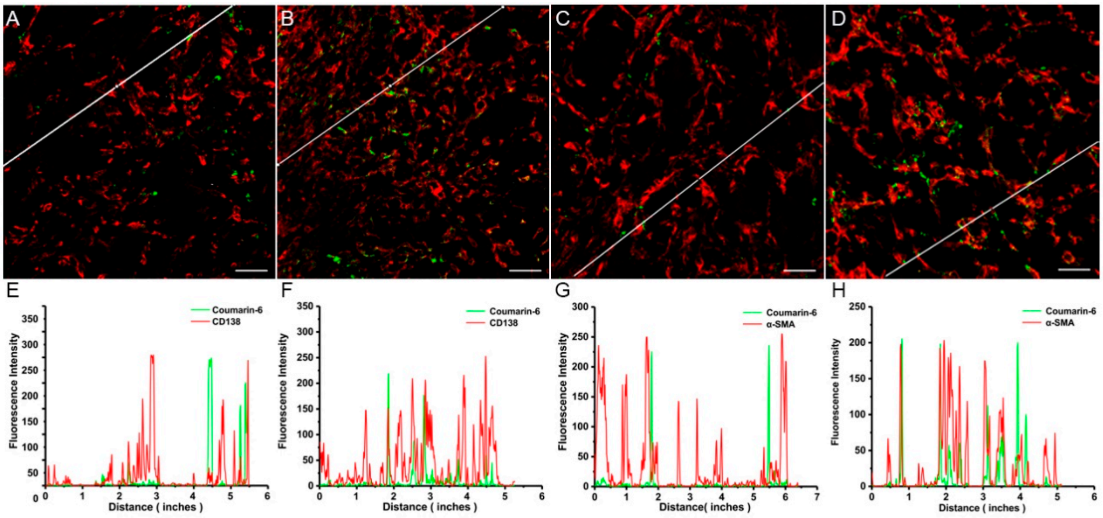

Figure S5. *In vivo* co-localized study of coumarin-6 labeled NPs (A, E, C, G) or CNPs (B, F, D, H) with fibroblasts (C, G, D, H) or myeloma cells (A, E, B, F) observed by confocal (A, B, C, D) and analyzed by ImageJ and origin (E, F, G, H). The signal intensity in the histogram is obtained from the line randomly put in the corresponding figure.

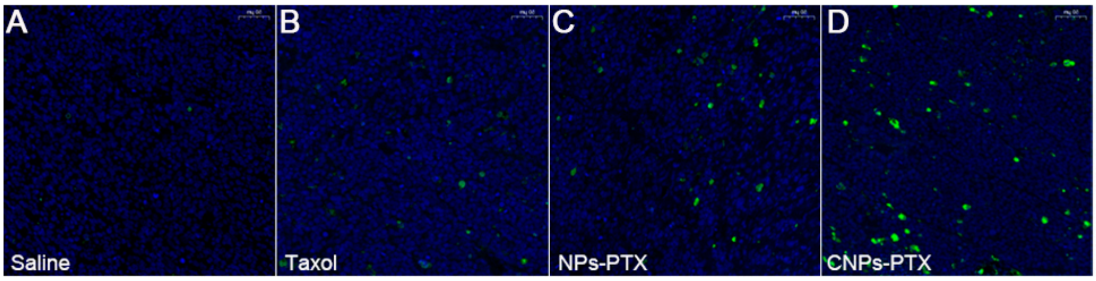

Figure S6. TUNEL staining of tumor tissue sections from MM bearing mice model of *in vivo* anti-myeloma study (left to right: Saline, Taxol, NPs-PTX, and CNPs-PTX).

Table S1. IC50 of RPMI8226 and NIH-3T3 by Taxol, NP-PTX and CNP-PTX calculated by GraphPad Prism 7.0 based on CCK8 assay.

IC50 (ng/ml)	Taxol	NPs-PTX	CNPs-PTX
RMPI 8226	16.36 ± 0.82 %	9.69 ± 1.15 %	6.86 ± 0.93 %
NIH-3T3	24.83 ± 3.14 %	20.40 ± 2.54 %	14.18 ± 1.22 %
